# Combination of OX40 Co-Stimulation, Radiotherapy, and PD-1 Inhibition in a Syngeneic Murine Triple-Negative Breast Cancer Model

**DOI:** 10.3390/cancers14112692

**Published:** 2022-05-29

**Authors:** Min Guk Han, Chan Woo Wee, Mi Hyun Kang, Min Ji Kim, Seung Hyuck Jeon, In Ah Kim

**Affiliations:** 1 Department of Radiation Oncology, Seoul National University Bundang Hospital, Seongnam-si 13620, Korea; han3270@snu.ac.kr (M.G.H.); biokmh@snu.ac.kr (M.H.K.); alswl6357@snu.ac.kr (M.J.K.); 2 Medical Science Research Institute, Seoul National University Bundang Hospital, Seongnam-si 13620, Korea; 3 Department of Tumor Biology and Cancer Research Institute, Seoul National University, Seoul 03080, Korea; 4 Department of Radiation Oncology, Seoul Metropolitan Government-Seoul National University Boramae Medical Center, Seoul 07061, Korea; wcw0108@snu.ac.kr; 5 Department of Radiation Oncology, Seoul National University College of Medicine, Seoul 03080, Korea; 6 Graduate School of Medical Science and Engineering, Korea Advanced Institute of Science and Technology, Daejeon 34141, Korea; hyck9004@naver.com; 7 Integrated Major in Innovative Medical Science, Seoul National University Graduate School, Seoul 03080, Korea

**Keywords:** radiotherapy, stereotactic body radiotherapy, immunotherapy, OX40, PD-1, breast cancer, immuno-oncology

## Abstract

**Simple Summary:**

This experimental study was designed in order to investigate the efficacy of the triple combination of radiation (SBRT), PD-1 blockade, and OX40 co-stimulation in a syngeneic murine model using ‘immunologically cold’ triple-negative breast cancer cells. SBRT can induce immunogenic tumor cell deaths and act as an in situ vaccine while OX40 signaling has been shown to improve anticancer immunity combined with PD-1 inhibition via multiple preclinical studies. In our study, triple combination therapy significantly improved primary/abscopal tumor control and reduced lung metastases compared to single or dual therapies. This was found to be through an increased ratio of CD8+ T cells to regulatory T cells and a reduced proportion of exhausted T cells in the tumor microenvironment.

**Abstract:**

Immune checkpoint inhibitors have been successful in a wide range of tumor types but still have limited efficacy in immunologically cold tumors, such as breast cancers. We hypothesized that the combination of agonistic anti-OX40 (α-OX40) co-stimulation, PD-1 blockade, and radiotherapy would improve the therapeutic efficacy of the immune checkpoint blockade in a syngeneic murine triple-negative breast cancer model. Murine triple-negative breast cancer cells (4T1) were grown in immune-competent BALB/c mice, and tumors were irradiated with 24 Gy in three fractions. PD-1 blockade and α-OX40 were administered five times every other day. Flow cytometric analyses and immunohistochemistry were used to monitor subsequent changes in the immune cell repertoire. The combination of α-OX40, radiotherapy, and PD-1 blockade significantly improved primary tumor control, abscopal effects, and long-term survival beyond 2 months (60%). In the tumor microenvironment, the ratio of CD8+ T cells to CD4 + FOXP3+ regulatory T cells was significantly elevated and exhausted CD8+ T cells (PD-1+, CTLA-4+, TIM-3+, or LAG-3+ cells) were significantly reduced in the triple combination group. Systemically, α-OX40 co-stimulation and radiation significantly increased the CD103+ dendritic cell response in the spleen and plasma IFN-γ, respectively. Together, our results suggest that the combination of α-OX40 co-stimulation and radiation is a viable approach to overcome therapeutic resistance to PD-1 blockade in immunologically cold tumors, such as triple-negative breast cancer.

## 1. Introduction

Breast cancers are typically immunologically cold tumors characterized by minimal T cell infiltration, a low tumor antigen expression, and an immunosuppressive tumor microenvironment (TME) although immunogenicity, the response to treatment, and the prognosis may differ according to the tumor subtype and tumor infiltrating lymphocytes [1,2]. This hampers the use of immune checkpoint inhibitors (ICIs) as standalone therapies for breast cancers. The triple-negative breast cancer (TNBC) subtype generally demonstrates the worst survival among breast cancer subtypes [3], and favorable outcomes are associated with elevated levels of tumor infiltrating lymphocytes in the TME [4,5,6]. Furthermore, TNBCs express higher levels of PD-L1 compared to luminal subtypes, which suggests that PD-1 blockade (PD-1B) may be effective in TNBC [7]. TNBCs also have a higher rate of somatic mutations that may generate tumor-specific neoantigens to prime the anti-tumor immune response [8]. Together, these findings indicate that some TNBC patients may benefit from the use of ICIs, such as atezolizumab and pembrolizumab [9,10,11], which have recently received clinical approval. Importantly, ICI treatment alone is insufficient to generate a response in TNBC [12,13], warranting the investigation of effective combination strategies.

Radiotherapy (RT) is a promising addition to ICI therapy due to its in situ vaccination effect on the TME. RT induces the release of tumor-associated antigens by provoking immunogenic tumor cell death. This leads to elevated major histocompatibility complex class I expression, upregulated type I interferon (IFN) signaling, the recruitment of antigen-presenting cells, and eventually the priming of anti-tumor cytotoxic T lymphocytes [14]. The favorable immune-modulating effects of RT are most profound when a hypofractionated form known as stereotactic body RT (SBRT) is used, and a dose range of 8–12 Gy per fraction is the most effective in preclinical models [15,16]. Of note, RT also induces immune suppressive cells, such as regulatory T cells (Tregs) [17]. As RT is an attractive candidate for therapeutic combination strategies in TNBC, overcoming its immunosuppressive side effects is an important area of investigation.

A promising solution to the immunosuppressive effects of RT is the type-I membrane glycoprotein OX40 (also known as CD134 or TNFRSF4), which is a member of the tumor necrosis factor receptor superfamily [18]. OX40 is a co-stimulatory molecule expressed on activated CD4+ and CD8+ T cells [18]. Importantly, OX40 signaling on effector T cells desensitizes them to the inhibitory effect of Tregs and increases memory cell expansion, the clonal proliferation of effector T cells, and the production of various stimulatory cytokines [19]. Alone or in combination with SBRT, PD-1B, or granulocyte-macrophage colony-stimulating factor gene-transfected tumor cell vaccine (GVAX), the co-stimulation of OX40 signaling with agonistic anti-OX40 monoclonal antibody (α-OX40) increases survival and tumor control in various preclinical models of breast cancer [20,21,22], glioblastoma [23,24], and lung cancer [25,26,27]. Although some combinations of α-OX40, SBRT, and PD-1B have been tested, no reports have evaluated the combination of all three in TNBC.

Here, we evaluated the efficacy of the triple combination of α-OX40, PD-1B, and RT in a syngeneic murine TNBC model. Our results also provide important insights into the mechanisms of interaction related to anti-tumor immunity.

## 2. Methods

### 2.1. Cell Line and Syngeneic Murine Tumor Model

Luciferase-tagged murine 4T1 (4T1-luc) or luciferase-untagged murine 4T1 (4T1) TNBC cells were stored, cultured, and prepared as previously described [28]. As luciferase may activate luciferase-specific CD8+ T cells [29,30], luciferase-untagged 4T1 cells were also included. Six hundred thousand 4T1-luc or 4T1-cells suspended in 100 μL of serum-free DMEM media were injected subcutaneously into the right hind limb (primary tumor) of 6-week-old female BALB/c mice (Orient Bio Inc., Seongnam-si, Korea). Cells were also injected into the left hind limb (secondary tumor) of the mice to evaluate abscopal effects. All animal experiments were performed under the approval of the Institutional Animal Care and Use Committee at the Clinical Research Institute, Seoul National University Bundang Hospital (IACUC No. BA-2011-308-104-03).

### 2.2. Radiation, PD-1 Blockade, and Anti-OX40 Co-Stimulation

Mice were randomized into eight groups (n = 5 for each group) as follows: control, α-OX40, PD-1B, RT; α-OX40 + PD-1B, α-OX40 + RT, PD-1B + RT, and α-OX40 + PD-1B + RT. For RT, mice were immobilized in a privately manufactured jig. A total of 24 Gy in three fractions, which mimics SBRT protocols for humans, was delivered to right hind limb tumors using 9-MeV electron beams (VitalBeam, Varian Medical Systems, Palo Alto, CA, USA) every 2 days starting on post-injection day 10. Left flank tumors were not irradiated to evaluate abscopal effects regardless of treatment group.

For α-OX40 co-stimulation, 100 µg of InVivoPlus anti-mouse OX40 (CD134) (BE0031, BioXCell, West Lebanon, NH, USA) was injected on post-injection days 11, 13, 15, 17, and 19 for a total dose of 500 µg. For PD-1B, 10 mg/kg of anti-mouse CD279 (GolnVivo^TM^, BioLegend, San Diego, CA, USA) was injected on post-injection days 10, 12, 14, 17, 19, and 21. All drugs were delivered intraperitoneally.

### 2.3. Tumor Size Measurement and Bioluminescence Imaging

Tumor size (length × width^2^ × 0.5) was measured every 2–3 days using a digital caliper (Digimatic Vernier Caliper, Mitutoyo Corp., Kawasaki, Japan). Bioluminescence images were obtained on post-inoculation day 24 as previously described [31].

### 2.4. Tissue Sample Preparation

Tumor, spleen, and lung tissues were extracted at the end of the study. Tumor and spleen tissues were harvested, minced with a scalpel blade, and incubated with 100 U/mL collagenase (Gibco, Grand Island, NY, USA) and 0.2 mg/mL DNase (Roche, Basel, Switzerland) in Hank’s Balanced Salt Solution for 30 min at 37 °C. Gross images of the extracted tumor and lung tissues were collected prior to any additional processing.

### 2.5. Immunohistochemistry

Immediately after harvest, a portion of the tumor tissue was fixed in 4% paraformaldehyde. Immunohistochemistry was performed on fixed tissues as previously described in detail [28]. The following primary antibodies were used: anti-CD4 (ab183685, Abcam, Cambridge, UK), anti-CD8 (ab203035, Abcam), anti-FOXP3 (NB100-39002, Novus Biologicals, Littleton, CO, USA), and anti-PD-L1 (ab2025921, Abcam). Images of stained slides were collected at a magnification of 40× using an Axioskop 40 light microscope (Carl Zeiss, Jena, Germany) and AxioVision 4.7 software. Optical density was quantified using ImageJ software ver. 1.8 (National Institutes of Health, Bethesda, MD, USA). The mean density of three sections per sample was calculated.

### 2.6. Flow Cytometric Analyses

Single-cell suspensions were prepared according to protocols for cell preparation for flow cytometry from Thermo Fisher Scientific. Briefly, samples were homogenized and then filtered through a cell strainer (93,100, SPL Life Sciences Co., Pocheon-si, Korea) to produce single-cell suspensions. Red blood cells were removed using ACK Lysing Buffer (A1049201, Gibco, Grand Island, NY, USA), and samples were washed and re-suspended in Cell Staining Buffer (420201, Biolegend, San Diego, CA, USA). After isolation of single cells, 1 × 10^6^ cells were aliquoted into tubes and stained using Fc blocker (156,604, Biolegend) to prevent nonspecific binding. Fluorochromes were conjugated to various antibodies, including CD3 (565,643; 555,274, BD Bioscience, San Jose, CA, USA), CD3 (100,217, Biolegend), CD4 (100,515, Biolegend), CD8b (550,798, BD Bioscience), CD11b (101,205; 101,207; 101,228; 101,212, Biolegend), CD11c (117,307, Biolegend), CD25 (102029, Biolegend), CD44 (103,011, Biolegend), CD45 (103,108; 103,106; 103,132; 103,112, Biolegend), CD62L (104,407; 104,431, Biolegend), CD103 (121,413, Biolegend), CD127 (135,021, Biolegend), CTLA-4 (106,315, Biolegend), FOXP3 (320,008, Biolegend), Granzyme B (372,211, Biolegend), Ki-67 (652,405, Biolegend), LAG-3 (125,209, Biolegend), PD-1 (135,213, Biolegend), and TIM-3 (134,011, Biolegend). For setting up the gating strategy, isotype controls were used for various antibodies, including FITC Isotype Ctrl Antibody (402,208, Biolegend), PE Isotype Ctrl Antibody (401,208, Biolegend), PerCP/Cyanine5.5 Isotype Ctrl Antibody (400,338, Biolegend), and APC Isotype Ctrl Antibody (402,206, Biolegend). Using these antibodies, immune cells were isolated from single-cell suspensions by flow cytometry (FACS; FACSCalibur, BD Biosciences) and analyzed using FlowJo software version 10 (Treestar, Ashland, OR, USA).

### 2.7. Measurement of Plasma Cytokines

Retro-orbital sinus blood was collected on post-injection days 7 and 24. Blood samples were centrifuged at 1000× *g* at 4 °C for 10 min to acquire plasma fractions. Plasma IFN-β (EPX01B-20644-901, Invitrogen™, Carlsbad, CA, USA), IFN-γ (EPX01A-20606-901, Invitrogen™), and TNF-α (EPXS010-20607-901, Invitrogen™) were measured according to the manufacturer’s instructions (MAN0016941, ProcartaPlex™ Multiplex Immunoassay, Invitrogen™). Briefly, plasma cytokine levels were measured using primary antibodies conjugated to magnetic beads and quantified on a Luminex™ 200 platform (Invitrogen™).

### 2.8. Cytometric Bead Arrays

Cytometric bead arrays were performed according to the commercially available platform protocol (BD Bioscience Cytometric Bead Array Mouse Th1/Th2/Th17 Cytokine Kit, 560,485). Briefly, 50 μL plasma (diluted 1:10), 50 μL beads, and 50 μL detection reagent were combined. The mixture was incubated for 2 h at room temperature and then washed by addition of 1 mL wash buffer and centrifugation at 200× *g* for 5 min. The supernatant was aspirated, and the beads were resuspended in 300 μL wash buffer and analyzed on a flow cytometer.

### 2.9. Statistical Analyses

PRISM statistical analysis and graphing software (GraphPad 8) was used to evaluate unpaired two-tailed Student’s *t*-tests or ANOVA in combination with Tukey’s or Dunnett’s multiple comparison tests. All results are presented as mean ± standard error. *p*-values under 0.05 were considered significant. Survival differences were evaluated by log-rank tests using the Statistical Package for Social Sciences, ver. 26.0 (IBM Corp., Armonk, NY, USA).

## 3. Results

### 3.1. Combination of RT, PD-1B, and α-OX40 Co-Stimulation Improves Primary Tumor Control, Enhances Abscopal Effects, Reduces Distant Metastasis, and Prolongs Survival

**Figure 1:**
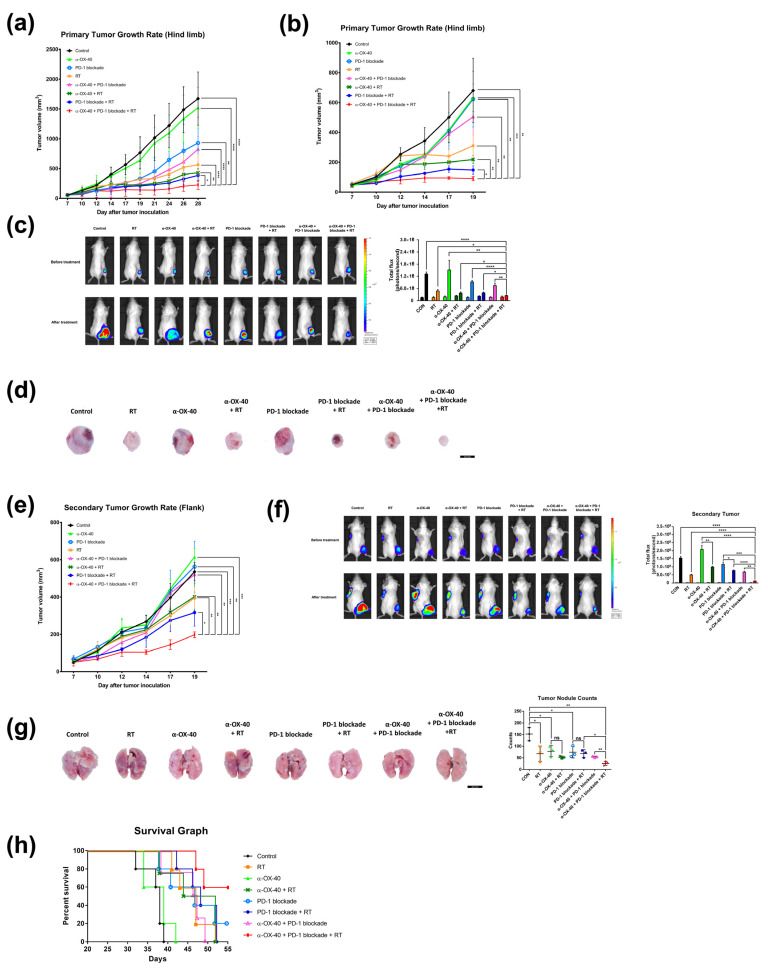
Combination of RT, α-OX40, and PD-1B increases primary and abscopal tumor control and prolongs survival. Primary tumor growth curves of subcutaneous (**a**) luciferase-tagged and (**b**) luciferase-untagged 4T1 implants in mice for each group: control, RT, α-OX40, PD-1B, α-OX40 + RT, PD-1B + RT, α-OX40 + PD-1B, and triple combination therapy (each group, n = 5). (**c**) Representative bioluminescence images of 4T1-luc tumor-bearing mice acquired before and after each treatment. Paired bar graphs comparing primary tumor volumes before and after treatment show total luminosity flux (emitted photons per second) for each group. (**d**) Images of extracted tumors from luciferase-untagged 4T1 tumor-bearing mice. (**e**) Secondary (abscopal) tumor growth curves in luciferase-untagged 4T1 tumor-bearing mice for each group (each group, n = 5). (**f**) Quantitation and representative bioluminescence images of 4T1-luc secondary tumor-bearing mice acquired before and after each treatment. (**g**) Metastatic lung nodules observed on post-implantation day 31. (**h**) Survival curves of 4T1-luc tumor-bearing mice for each treatment group (each group, n = 5). * *p* < 0.05, ** *p* < 0.01, *** *p* < 0.001, **** *p* < 0.0001. Abbreviations: 4T1-luc, luciferase-tagged 4T1; CON, control; RT, radiotherapy.

Triple combination therapy resulted in significantly superior tumor control of the primary tumor compared to all other groups (all *p* < 0.05) for both luciferase-tagged and luciferase-untagged 4T1 tumors (Figure 1a,b). This was also confirmed by in vivo bioluminescence imaging (all *p* < 0.05) (Figure 1c) and gross examination (Figure 1d) of 4T1-luc tumors.

Furthermore, triple combination significantly inhibited growth of the unirradiated secondary tumors implanted at the left flank (all *p* < 0.05) compared to all other treatment arms, reflecting the “abscopal effect” of RT (Figure 1e,f). IVIS imaging confirmed significantly enhanced abscopal effects in mice treated with RT compared to mice in groups that did not receive RT, namely the control (*p* < 0.0001), α-OX40 (*p* = 0.0026), PD-1B (*p* = 0.0410), and α-OX40 + PD-1B (*p* = 0.0053) groups (Figure 1f).

In addition to primary and secondary tumor response, we also evaluated distant metastases and overall survival. We found significantly fewer metastatic lung nodules in the triple combination group (Figure 1g). Triple combination therapy resulted in significantly fewer numbers of lung nodules compared to any dual combination therapy (α-OX40 + RT, *p* = 0.0117; PD-1B + RT, *p* = 0.0174; α-OX40 + PD-1B, *p* = 0.0061). Additionally, the median survival of 55 days was only met by the triple combination group (Figure 1h). All mice died during follow-up in the RT + PD-1B group, whereas 60% of mice were alive at the end of follow-up in the triple combination group. Survival improved with addition of α-OX40 (*p* = 0.0897) but did not reach significance. The addition of RT significantly improved survival in mice treated with α-OX40 + PD-1B (*p* = 0.0320). Based on the substantial improvement in tumor, metastasis, and survival outcomes with triple combination therapy, we went on to evaluate the molecular basis for these effects.

### 3.2. Anti-Tumor Immunomodulatory Effects of Anti-OX40 Co-Stimulation and RT in the Spleen

**Figure 2:**
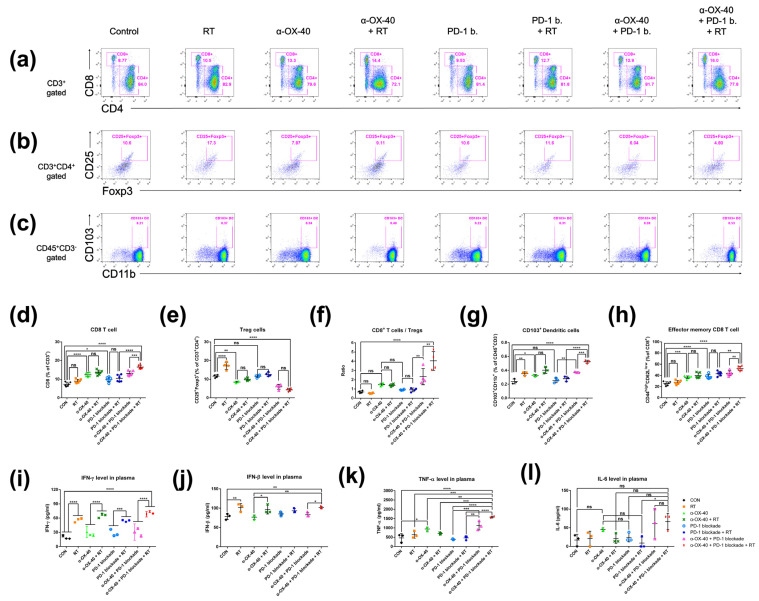
Immune cell profiles in the spleen. Representative flow cytometry plots for (**a**) CD4+ or CD8+ T cells, (**b**) CD4 + FOXP3+ Tregs, and (**c**) CD45 + CD103+ dendritic cells. (**d**–**h**) Cell population percentages calculated from flow cytometry analyses. ELISpot quantification of (**i**) IFN-γ, (**j**) IFN-β, and (**k**) TNF-α plasma levels. (**l**) Cytometric bead array results for IL-6 plasma levels. * *p* < 0.05, ** *p* < 0.01, *** *p* < 0.001, **** *p* < 0.0001. Abbreviations: CON, control; RT, radiotherapy; Treg, regulatory T cell; DC, dendritic cell; ns, not significant.

We compared the immune repertoire between treatment groups by assessing CD8+ T cells, CD4 + FOXP3+ Tregs, the CD8+ T cell to Treg ratio, CD45 + CD11b + CD103+ dendritic cells, and CD44^high^CD62L^low^ effector memory CD8+ and CD4+ T cells in spleen samples from 4T1-luc tumor-bearing mice (Figure 2). Compared to mice treated without α-OX40 or RT, the addition of α-OX40 or RT significantly increased the number of CD8+ T cells in spleen tissues (Figure 2a,d,f). Regardless of the treatment arm, the addition of α-OX40 reduced the number of Tregs (Figure 2b,e,f) and increased the number of effector memory CD8+ T cells in spleen tissues (Figure 2h), which resulted in the significant elevation of the CD8+ to Treg ratio (Figure 2f). Although the number of Tregs were significantly increased in the RT group compared to the control group, we did not observe any significant increase in Tregs when RT was combined with α-OX40 ± PD-1B. The number of dendritic cells, which favor the CD8+ T cell response [32,33], significantly increased with α-OX40 (control vs. α-OX40; PD-1B vs. PD-1B + α-OX40; PD-1B + RT vs. triple combination), and the triple combination demonstrated the highest dendritic cell response (Figure 2g).

Plasma IFN-γ and IFN-β levels were significantly increased by RT, whereas α-OX40 and PD-1B had minimal effects (Figure 2i,j). The increase in IFN-β levels was not eminent only when RT was added to PD-1B monotherapy. TNF-α levels were significantly elevated in the triple combination group (Figure 2k). The levels of IL-6, a cytokine known to inhibit TGF-β-induced Treg differentiation, were also higher in the two groups treated by α-OX40 and PD-1B with or without RT, although it did not reach statistical significance (Figure 2l) [34,35]. Although α-OX40 and RT had inconsistent effects on the CD4+ T cell population in spleen tissues (Supplementary Figure S1a), the addition of α-OX40 to either RT or PD-1B significantly increased the number of effector memory CD4+ T cells observed in the spleen (Supplementary Figure S1b).

### 3.3. Anti-Tumor Immunomodulatory Effects of α-OX40 Co-Stimulation and RT in the TME

**Figure 3:**
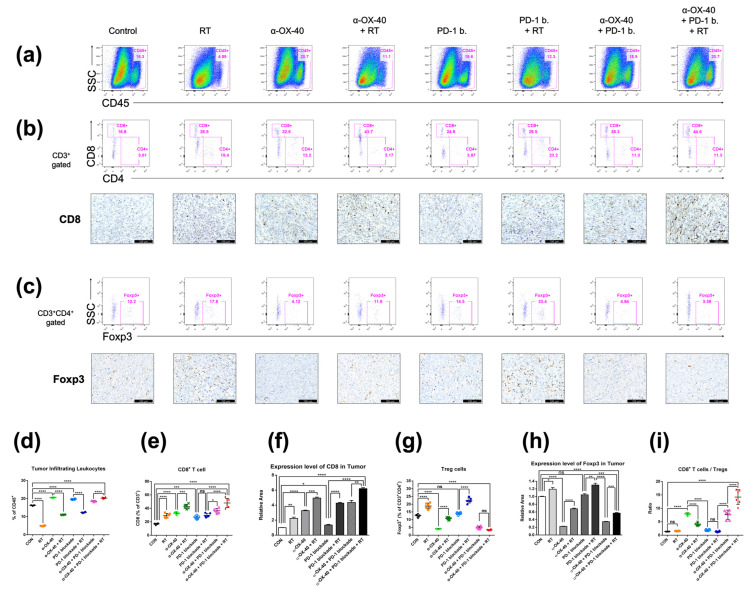
Immune cell profiles in the tumor microenvironment. Representative flow cytometry plots and immunohistochemistry images of (**a**) CD45+ tumor-infiltrating leukocytes, (**b**) CD4+ or CD8+ T cells, and (**c**) CD4 + FOXP3+ Tregs. (**d**–**i**) Cell population percentages calculated from flow cytometry analyses and immunohistochemistry. * *p* < 0.05, ** *p* < 0.01, *** *p* < 0.001, **** *p* < 0.0001. Abbreviations: CON, control; RT, radiotherapy; Treg, regulatory T cell; ns, not significant.

Flow cytometry and immunohistochemistry demonstrated similarly favorable immunomodulatory effects of α-OX40 and RT in the TME (Figure 3). The treatment of α-OX40 or PD-1B significantly increased the number of tumor-infiltrating leukocytes but not RT (Figure 3a). The addition of α-OX40 or RT significantly increased the number of tumor-infiltrating CD8+ T cells, and triple combination therapy demonstrated the highest level of CD8+ T cell infiltration (Figure 3b,e,f,i). Importantly, the immunosuppressive Treg population was lowest in tumors treated with α-OX40 regardless of the combination treatment (Figure 3c,g–i). Despite the significant increase in FOXP3 expression when RT was added to each treatment (Figure 3h), we did not observe a significant increase in TME Tregs in the triple combination group (Figure 3g). This finding might be due to the increase in the total number of tumor-infiltrating leukocytes in the triple combination group compared to other groups treated with RT (Figure 3d). Compared to spleen tissues, triple combination therapy resulted in a dramatic increase in the CD8+ T cell to Treg ratio (Figure 3i). The addition of α-OX40 to any treatment similarly increased the CD8+ T cell to Treg ratio in the TME.

### 3.4. Triple Combination Therapy Results in Less Exhausted Tumor-Infiltrating CD8+ T Cells

**Figure 4:**
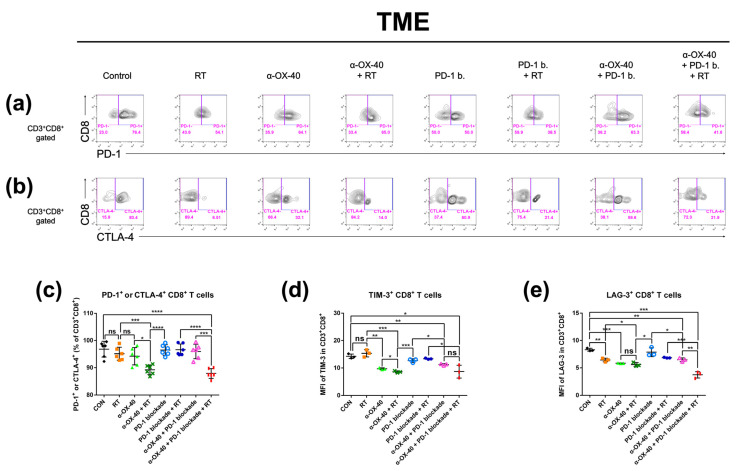
Expression of exhaustion markers on CD8+ T cells in the tumor microenvironment. The percentage of CD8+ T cells expressing exhaustion markers (PD-1, CTLA-4, TIM-3, and LAG-3) was calculated by flow cytometry. Representative flow cytometry plots of (**a**) PD-1+ or PD-1- and (**b**) CTLA-4+ or CTLA-4- on CD8+ T cells. Results are presented as (**c**) percentage of T cells expressing PD-1 and/or CTLA-4, (**d**) MFI of TIM-3 in CD8+ T cells, and (**e**) MFI of LAG-3 in CD8+ T cells. * *p* < 0.05, ** *p* < 0.01, *** *p* < 0.001, **** *p* < 0.0001. Abbreviations: MFI, mean fluorescent intensity; CON, control; RT, radiotherapy; ns, not significant.

Exhausted T cells in the TME represent an immunologically hyporesponsive state due to prolonged exposure to antigens. Thus, we investigated CD8+ tumor-infiltrating T cells for signs of exhaustion and compared the proportion of exhausted T cells between therapy groups. PD-1, CTLA-4, TIM-3, and LAG-3 expressions were evaluated as markers of T cell exhaustion by flow cytometry (Figure 4) [36]. The proportion of PD-1 and/or CTLA-4 expressing CD8+ T cells was significantly lower when α-OX40 and RT were combined (Figure 4c). Furthermore, TIM-3 expressing CD8+ T cells were significantly reduced when mice were treated with α-OX40 regardless of other treatments (Figure 4d). Triple combination therapy demonstrated the lowest proportion of LAG-3 expressing CD8+ T cells (Figure 4e).

### 3.5. α-OX40 Therapy Increases the Proportion of Proliferative Tumor-Infiltrating T Cells

**Figure 5:**
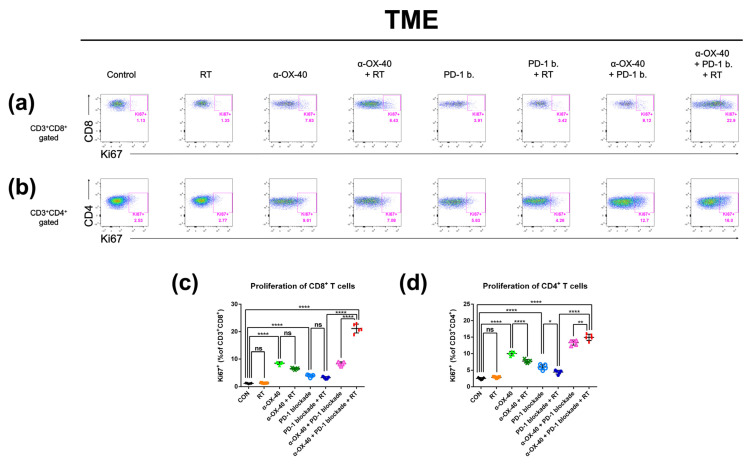
Proliferative T cell compartments in the tumor microenvironment. Representative flow cytometry plots of (**a**) CD8 + Ki67+ and (**b**) CD4 + Ki67+ T cells. The percentage of T cells expressing the proliferative marker Ki-67 in (**c**) CD8+ and (**d**) CD4+ T cell populations in the tumor microenvironment was calculated by flow cytometry. * *p* < 0.05, ** *p* < 0.01, *** *p* < 0.001, **** *p* < 0.0001. Abbreviations: CON, control; RT, radiotherapy; ns, not significant.

As we observed improved tumor infiltration of T cells with α-OX40, we evaluated the proportion of proliferating CD8+ and CD4+ T cells using expression of the proliferation marker Ki-67. In the TME, the addition of α-OX40 significantly enhanced the Ki-67 + CD8+ T cell population regardless of treatment (Figure 5c). Although we did not observe a significant effect in the absolute number of CD4+ T cells in the TME (Supplementary Figure S1c), the proportion of proliferating CD4+ T cells was significantly increased by the addition of α-OX40 in the TME (Figure 5d). The proportions of Ki-67 + CD8+ and Ki-67 + CD4+ T cells were highest in tumors treated with triple combination (Figure 5c,d).

## 4. Discussion

ICIs targeting PD-1, PD-L1, and CTLA-4 have rapidly become a major cancer treatment option over the past decade. The recent success of ICIs has also highlighted the immune susceptibilities of early-stage and metastatic TNBCs [9,10,11]. However, most patients with metastatic TNBC demonstrate a suboptimal survival and a poor response to ICI monotherapy, even in patients with PD-L1+ tumors [12,13]. Therefore, combination strategies that complement the insufficient anti-tumor immune response in TNBC warrant investigation.

To overcome the limited efficacy of PD-1B monotherapy in TNBC, we evaluated the combination of α-OX40, RT, and PD-1B, which has not been tested elsewhere, in a syngeneic murine 4T1 TNBC model. Triple combination therapy significantly improved tumor control of the irradiated primary tumor as well as the non-irradiated secondary tumor in both 4T1-luc and luciferase-untagged 4T1 tumors, compared to the controls, monotherapies, and dual therapies (Figure 1). Long-term survival was also superior in the triple combination group (Figure 1h).

The high-dose hypofractionated RT schedule used in the current study, “SBRT,” is known for its high local control rate, ranging from 75–90%, and its potential survival benefit in various oligometastatic tumor types [37,38]. In our preclinical study, the tumor control rate of SBRT was further enhanced by the addition of α-OX40 and PD-1B when compared to RT alone, RT + α-OX40, or RT + PD-1B (Figure 1a–d). Enhanced tumor control was accompanied by increased tumor-infiltrating CD8+ T cells, reduced immunosuppressive Tregs, elevated CD8+ T cell to Treg ratios, increased functional non-exhausted CD8+ T cells, and increased proliferative CD8+ T cells in the TME with the addition of α-OX40 (Figure 3 and Figure 4). Co-stimulation with α-OX40 induces expansion of effector T cells and inhibits the natural and inducible Treg function [19]. Similar results have been reported in preclinical studies of α-OX40 co-stimulation. For example, in anti-PD-1 resistant 344SQ metastatic lung cancer cells, the addition of intratumoral α-OX40 following SBRT (36 Gy in three fractions) improves local control, abscopal effects, and survival by augmenting CD8+ T cell expansion and infiltration in the TME [27]. More specifically, CD8+ T cells in the TME significantly increased in α-OX40 and α-OX40 + RT groups when compared to control or RT groups, respectively. Another study reported improved survival in a Lewis lung carcinoma model when intratumoral α-OX40 was added to SBRT (20 Gy in one fraction) [26]. OX40 + CD8+ T cells were augmented in the draining lymph nodes of the lung tumors by α-OX40 [26]. In a syngeneic pancreatic cancer (Panc02) model, a significant increase in tumor control and cytotoxic T cells occurs in the TME when α-OX40 is added to RT and hyperthermia [39]. The addition of α-OX40 is also consistently reported to reduce the presence of Tregs in the TME. In GL261 glioma cells, a significant reduction in the intra-glioma Treg subpopulation is achieved when α-OX40 is added to GVAX or to PD-1B + GVAX therapy [23,24]. One of the few downsides of using RT in anti-tumor immunity strategies is the increase in Tregs in the TME [40], which was also observed in our study (Figure 3e,f). Our results suggest that the negative effect of RT can be abrogated by the addition of α-OX40, which successfully reduced the number of Tregs and increased the number of effector T cells in our model, resulting in a favorable anti-tumor immune repertoire (Figure 3e,f).

T cells are a major component of adaptive anti-tumor immunity. RT contributes to anti-tumor immunity by inducing immunogenic cell death, which generates tumor antigens that are recognized by T cells. After exposure to tumor antigens, T cells gain effector functions and produce cytokines, granzymes, and perforin that have additional cytotoxic effects on tumor cells. However, prolonged antigen stimulation and the complex immunosuppressive network of the TME can induce a dysfunctional state in T cells, which results in T cell exhaustion. As a result, even T cells that successfully expand and infiltrate the TME may be functionally limited by exhaustion [39]. Thus, T cell exhaustion is an important consideration when designing immunomodulatory therapy combinations.

We found that the combination of α-OX40 and RT with or without PD-1B significantly reduced the proportion of CD8+ T cells expressing PD-1 and/or CTLA-4 in the TME (Figure 4). TIM-3+ and LAG-3+ CD8+ T cells were also significantly reduced by α-OX40, indicating that α-OX40 co-stimulation with or without RT prevents effector T cells from becoming exhausted. Although PD-1B itself restores the function of these exhausted CD8+ T cells [41], combination therapy with α-OX40 and RT further reduced the proportion of exhausted T cells in our study, supporting an immunological rationale for this triple combination strategy. Similarly, α-OX40 is reported to reverse intracranial T cell exhaustion, evidenced by PD-1, TIM-3, or LAG-3 expression in GL261 glioma cells [24]. In breast cancer cells, α-OX40 has been reported to rescue effector T cell dysfunction following MEK inhibition [42]. Our findings are consistent with these studies and extend the use of α-OX40 therapy to combination strategies that further enhance anti-tumor immune effects.

Notably, our triple combination strategy also improved abscopal effects in unirradiated tumors and inhibited gross lung metastases, indicating an enhanced systemic anti-tumor immune response with the triple combination (Figure 1). Consistent with our findings in the TME, we observed increased CD8+ T cell to Treg ratios in the splenocytes of mice treated with α-OX40, and which was highest in the triple combination group (Figure 2). Elevated numbers of dendritic cells, which play a major role in activating effector T cells, were also present in the splenocytes of RT-treated mice, and the highest levels of dendritic cells were found in the triple combination group (Figure 2). OX40 co-stimulation upregulates Batf3 [43], which drives the expansion of dendritic cells and initiates a CD8+ T cell response [33]. To evaluate the CD8+ T cell response, we measured the plasma levels of cytokines produced by activated CD8+ T cells. However, although we observed an increase in abscopal tumor control of secondary tumors in mice treated with RT compared to mice treated with the respective treatment without RT (Figure 1e), we did not observe a significant increase in CD8+ T cells, CD8+ T cell to Treg ratios, and dendritic cells in splenocytes expected for mice treated with triple combination. Instead, what we found was a significant increase in plasma IFN-γ when RT was added to each treatment (Figure 2i). Plasma IFN-γ, a major cytokine predominantly produced by activated lymphocytes such as CD8+ T cells, was significantly increased by the addition of RT to the primary tumor regardless of other treatments (Figure 2i). Notably, IFN-γ is crucial to the tumor-eliminating processes of CD8+ cytotoxic T cells and may contribute to the observed anti-tumor effects in our study [44]. Therefore, although there was no significant change in the number of CD8+ T cells systemically, we can assume an increase in activated CD8+ T cells systemically. The significant reduction of lung metastases in the triple combination group might have been the consequences of the effectively controlled primary tumor. Indeed, although not statistically significant, the mean number of gross lung nodules were reduced when RT was added (results not shown) to each treatment correlating with the enhanced primary tumor control by RT (Figure 1a,b). RT also elevated IFN-β levels, which is another crucial perquisite for RT-mediated adaptive anti-tumor immunity [15]. The proliferation of CD8+ and CD4+ T cells increased with the combination of PD-1B and α-OX40 in the TME. Our results suggest that the systemic effect of α-OX40 and RT combined with overall effector CD8+ T cell priming and proliferation synergize to enhance local and systemic tumor control with the greatest impact achieved by triple combination therapy.

Although our preclinical data indicate that the combination of RT, PD-1B, and α-OX40 is a promising therapeutic strategy in “cold” TNBC, there are several issues to address before clinical adoption. First of all, the number of mice in each group was small in our study making the small random errors or effects to have an apparent effect on the results. Furthermore, the appropriate doses of α-OX40 or PD-1B must be determined for humans and the most effective order of the treatments must be identified. Although more desirable outcomes have been observed when α-OX40 is delivered prior to PD-L1 or following RT [22,25,26,27,39], the triple combination of these treatments has not been tested. Immune-related adverse events are also a concern and can occur when combining different immunotherapeutic agents [45]. While OX40 being mainly expressed on activated CD8+ and CD4+ T cells, there is difference in OX40 expression on Tregs between mice and human. In humans, OX40 expression is higher in Tregs isolated from inflammatory sites such as tumors compared to Tregs in the peripheral blood [46], which imply that α-OX40 treatment is a reasonable approach in terms of immune-related side effects in sites other than the tumor. Furthermore, a possible dual function of OX40 stimulation of expanding Treg population in non-tumor tissues might help to further limit autoimmune toxicity associated with immunotherapy [47]. In our study, we did not observe any serious toxicity with intraperitoneal delivery of 500 µg (100 µg per injection) α-OX40 and 60 mg/kg (10 mg/kg per injection) PD-1B based on gross examination and body weight of mice (Supplementary Figure S2). As these are broad indicators of toxicity, more detailed toxicity profiles and hematologic markers should be investigated. The ongoing clinical trials of α-OX40 agonist in various combinations would firmly address the toxicity profiles in human [48]. The expansion of CD8+ T cells also needs to be validated to determine whether this was a result of tumor-specific immune effects.

## 5. Conclusions

In summary, we demonstrated that the combination of RT, PD-1B, and α-OX40 improves local and systemic tumor control as well as survival. We attribute this finding to a favorable balance of CD8+ T cells and Tregs in the TME. We suggest that the combination of RT, PD-1B, and α-OX40 is a viable strategy for overcoming the therapeutic resistance of immunologically cold tumors, such as TNBC, to ICIs.

## Data Availability

The datasets used and/or analysed during the current study are available from the corresponding author on reasonable request.

## Supplementary Materials

The following supporting information can be downloaded at: https://www.mdpi.com/article/10.3390/cancers14112692/s1. Figure S1: CD4+ T-cell profiles in the spleen and tumor microenvironment, Figure S2: Mice body weight after treatment.
