# Supplementary material for: Combination of OX40 Co-Stimulation, Radiotherapy, and PD-1 Inhibition in a Syngeneic Murine Triple-Negative Breast Cancer Model
Source: Cancers (Basel). 2022 May 29;14(11):2692. doi: 10.3390/cancers14112692 (PMC9179485; doi:10.3390/cancers14112692)

Supplementary S1. CD4+ T-cell profiles in the spleen and tumor microenvironment. Percentage of **(a)** CD4+ and **(b)** effector memory CD4+ T-cell population in spleen is plotted from each flow cytometry analysis. **(c)** Immunohistochemistry results for CD4+ T cells in the tumor microenvironment. *P < 0.05, **P < 0.01, ***P < 0.001, ****P < 0.0001, and ns = not significant. **Abbreviations**: CON, control; RT, radiation; PD-1B, PD-1 blockade.

(a)

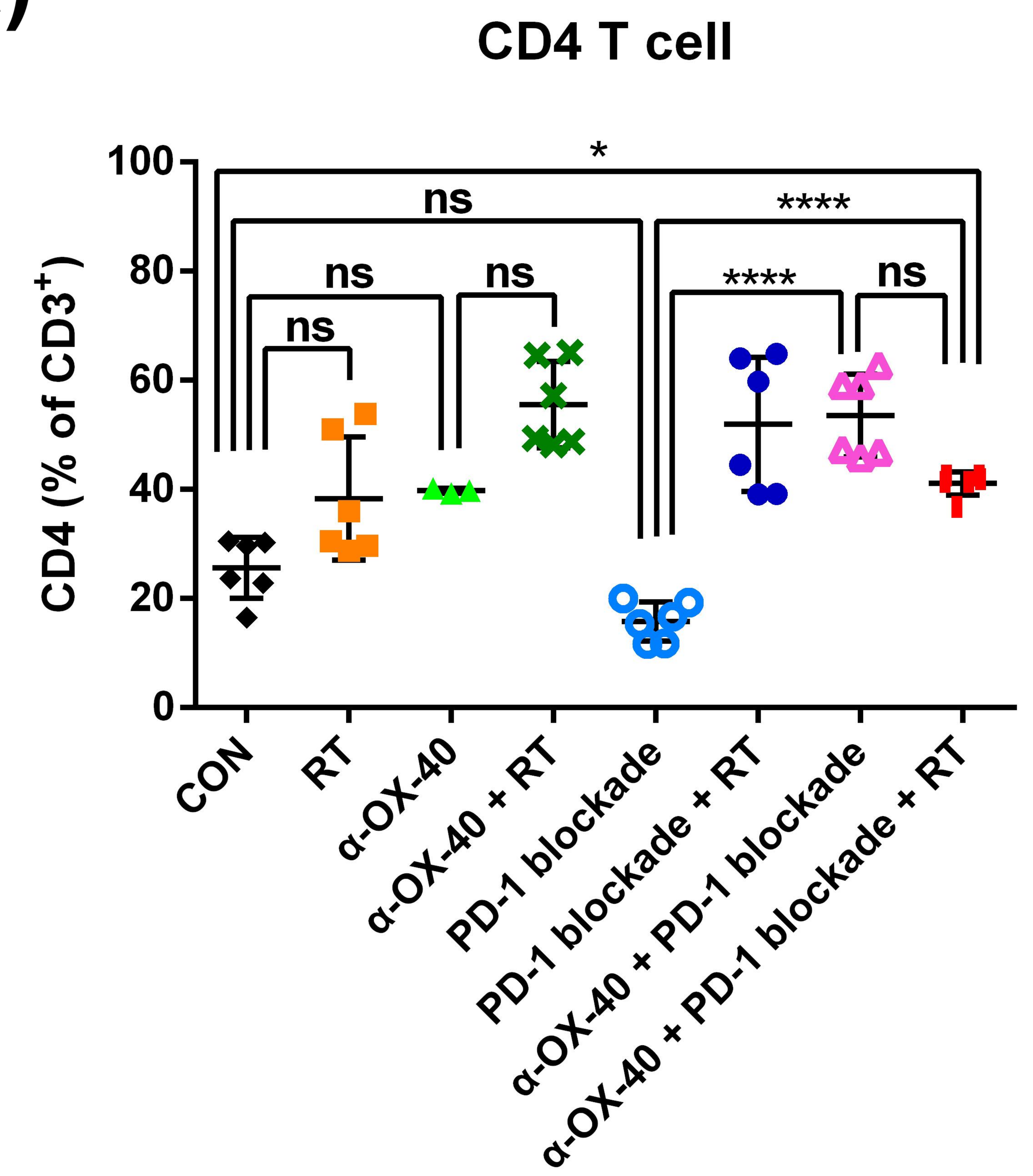

(b)

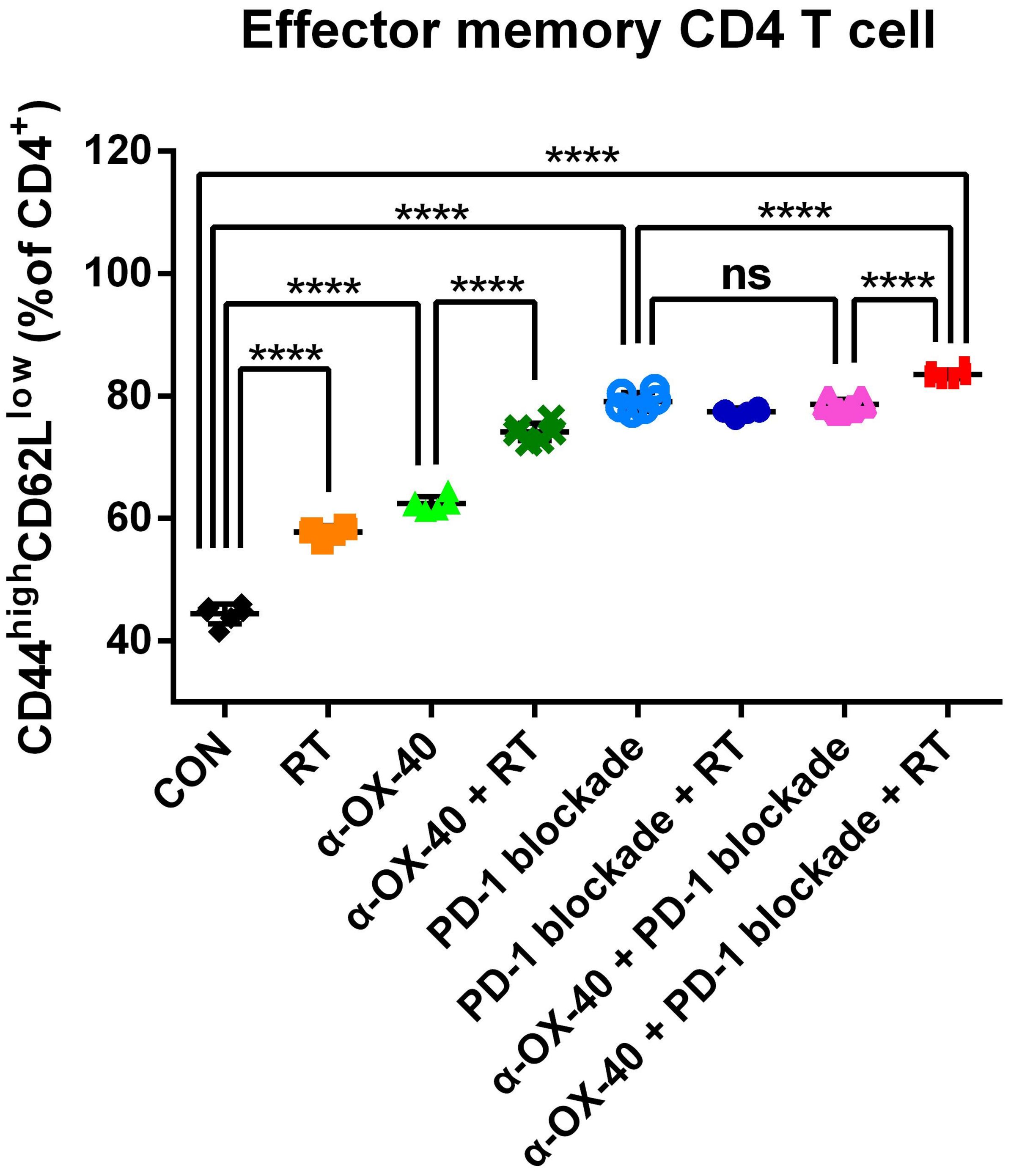

(c)

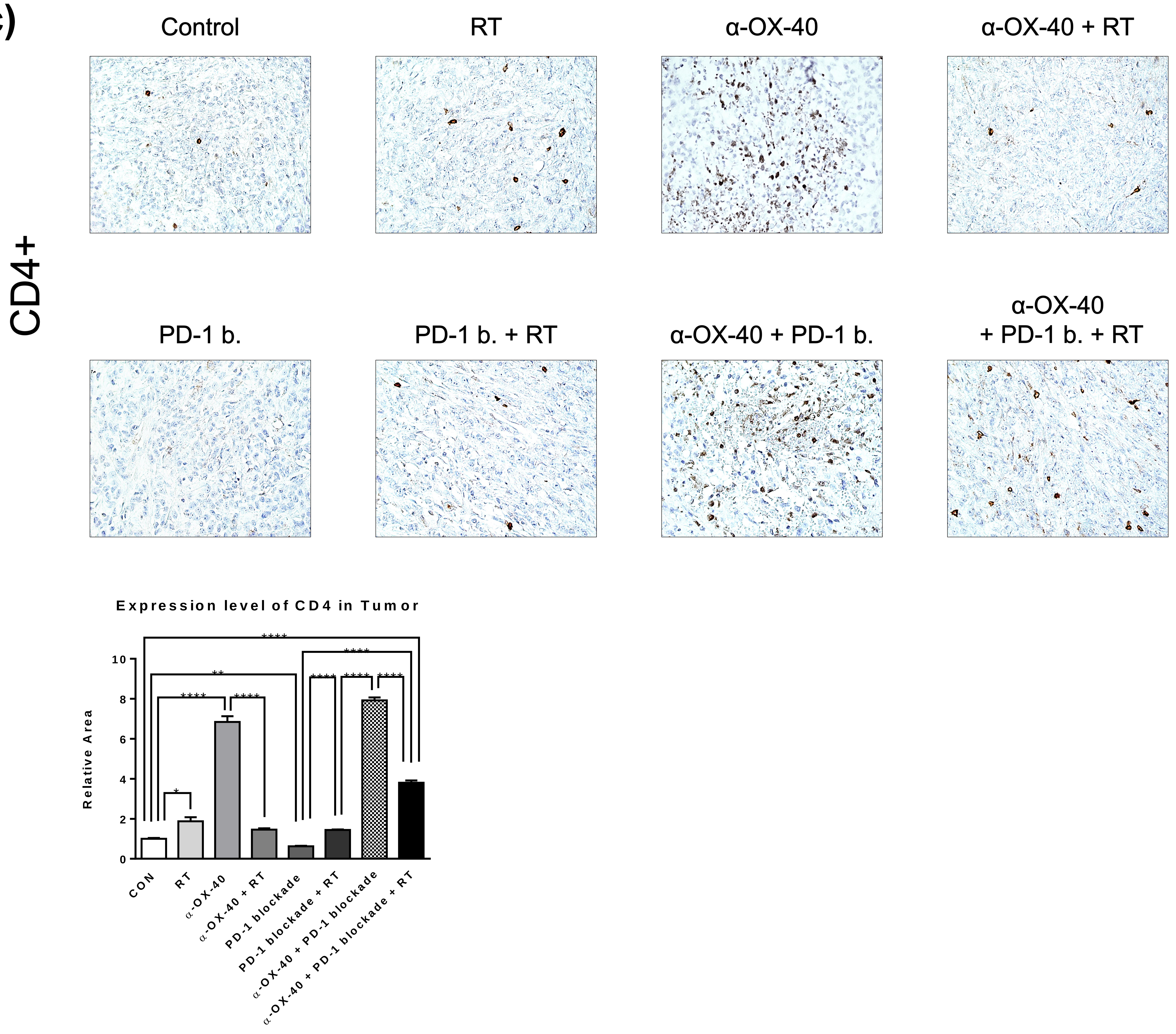

Supplement: Supplementary file 1 [file cancers-14-02692-s001.zip › Supplementary Figure S1.pdf]
