# Supplementary material for: Combination of OX40 Co-Stimulation, Radiotherapy, and PD-1 Inhibition in a Syngeneic Murine Triple-Negative Breast Cancer Model
Source: Cancers (Basel). 2022 May 29;14(11):2692. doi: 10.3390/cancers14112692 (PMC9179485; doi:10.3390/cancers14112692)

Supplementary S2. Mice body weight after treatment.

Body weight of tumor-bearing mice treatment was measured every other day to evaluate treatment after treatment. Abbreviations: RT, radiation.

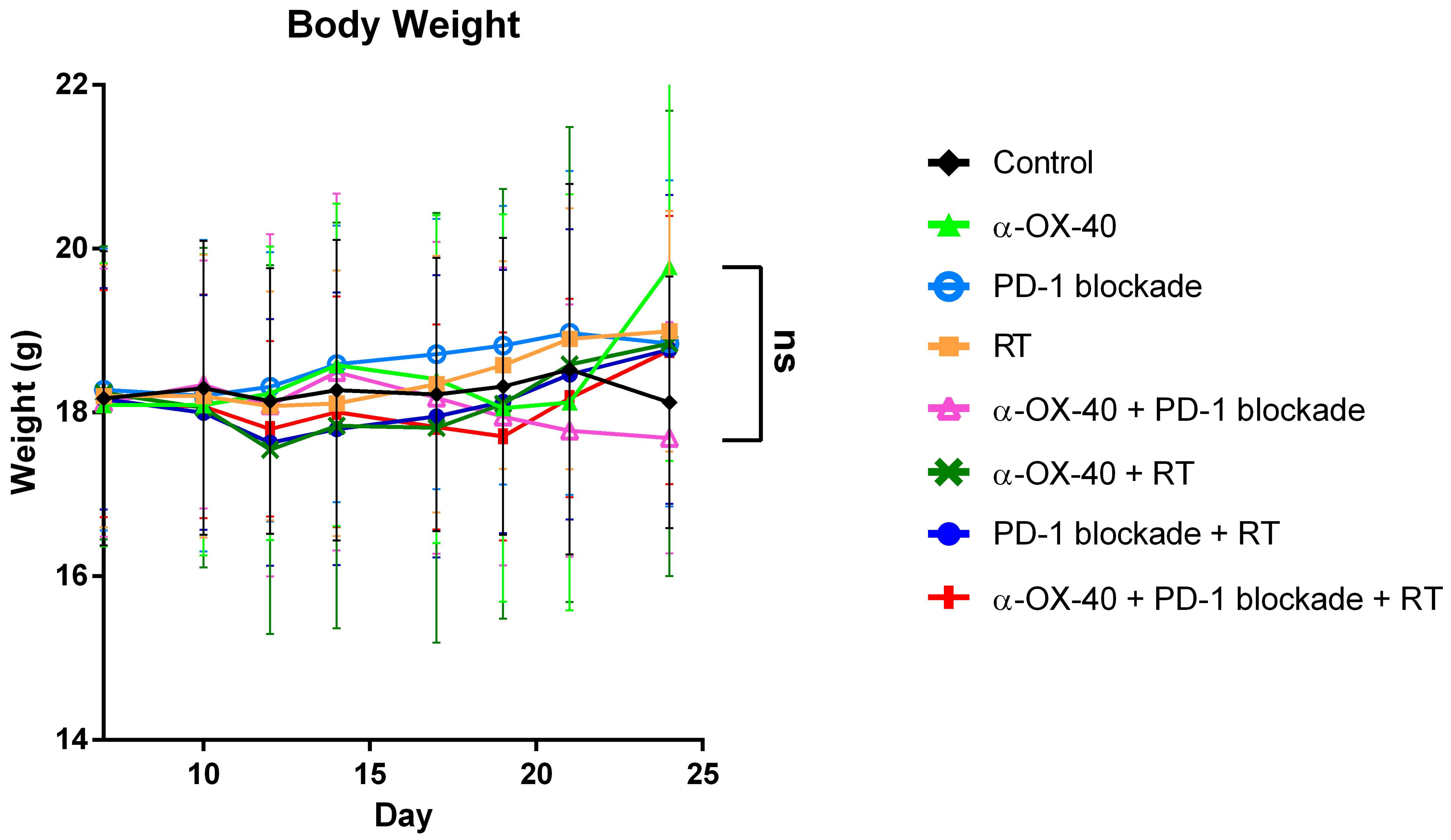

Supplement: Supplementary file 1 [file cancers-14-02692-s001.zip › Supplementary Figure S2.pdf]
